# Anatomical Basis for the Mobility of the Esophagus: Implications for Catheter Ablation of Atrial Fibrillation

**Published:** 2008-02-01

**Authors:** Subramaniam C Krishnan, Miguel Salazar, Navneet Narula

**Affiliations:** University of California at Irvine, School of Medicine, 101 The City Drive, Building 53, Orange, CA 92868

**Keywords:** ablation, atrium, fistula, injury, pathology

## Abstract

We present autopsy data from a patient that illustrates the anatomical factors that allow the esophagus to be a mobile structure, especially with respect to the posterior left atrial wall.

## Background

Esophageal injury and the development of an atrio-esophageal fistula is a devastating complication of percutaneous and surgical ablation therapy for atrial fibrillation [1]. The potential for this complication has led to greater attention to the anatomy of the esophagus and its relationship to the posterior left atrium. The esophagus and posterior left atrial wall are in close contact over a wide area that often encompasses the ablation sites. The anatomic location of the esophagus exhibits marked variability. Even during a single procedure, the esophagus is very mobile and  can spontaneously shift by more  than  4 cms with respect to the posterior atrium [2]. Based on this observation, some investigators have tried to manually move the esophagus  away from a desired ablation location [3]. This technique is receiving increasing attention. Autopsy data is presented below, which illustrates anatomical factors that permit this esophageal mobility. The study was approved by the institutional review board at our hospital.

## Case/autopsy presentation

**Figure 1:**
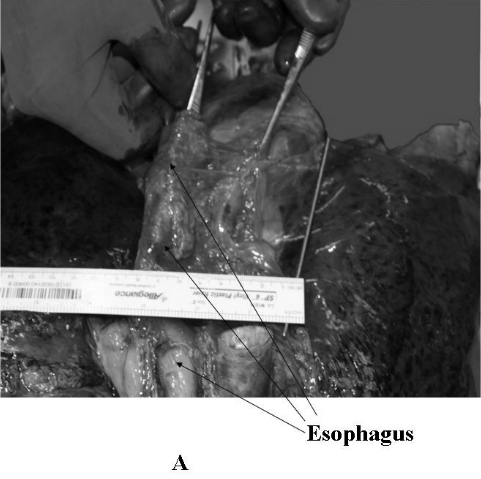

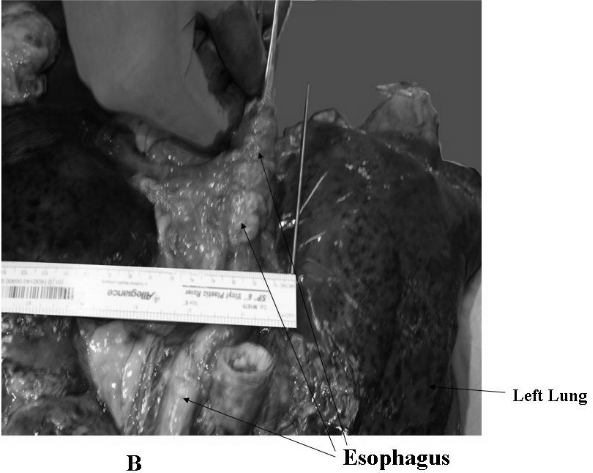
A dorsal view of the lungs and mediastinum is shown. The blunt end of a forceps held with the right hand of the author is placed within the lumen of the esophagus and the structure is moved from right (panel A) to left (panel B). The parietal pericardium overlying the posterior wall of the left atrium is held fixed with a pair of toothed forceps (held with the left hand). The esophagus is seen to be displaced by approximately 7 cms with respect to the parietal pericardium.

**Figure 2:**
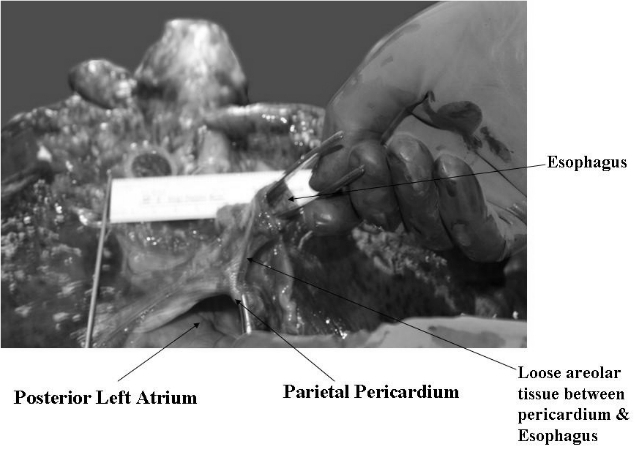
A caudal view of the lungs and mediastinum is seen. The parietal pericardium overlying the posterior left atrium is held fixed by a pair of toothed forceps. Lax areolar/fatty tissue is seen between the esophagus and the parietal pericardium and the laxity of this tissue accounts for the extreme mobility of the esophagus with respect to the parietal pericardium.

A 60 year old male patient who died of noncardiac causes underwent an autopsy. He had no history of heart or esophageal disease and there was no prior history of cardiac or mediastinal surgery. A dorsal view of the lungs and mediastinum is shown in Figure 1. The blunt end of a forceps held with the right hand of the author is placed within the lumen of the esophagus and the structure is moved from right (panel A) to left (panel B).  The parietal pericardium overlying the posterior wall of the left atrium is held fixed with a pair of toothed forceps (held with the left hand). The esophagus is seen to be moved by approximately 7 cms with respect to the parietal pericardium. Figure 2 shows a caudal view illustrating the presence of loose areolar tissue that allows for mobility of the esophagus with respect to the parietal pericardium. The parietal pericardium overlying the posterior left atrium is held fixed by a pair of toothed forceps. Lax areolar/fatty tissue is seen between the esophagus and the parietal pericardium and the laxity of this tissue accounts for the extreme mobility of the esophagus with respect to the parietal pericardium. 

These figures are shown to illustrate the anatomical basis for the marked mobility of the esophagus that can be seen during left atrial ablation procedures. It appears that there is loose fatty areolar tissue that connects the esophagus to the pericardium overlying the posterior atrial wall and the laxity of this tissue accounts for the marked mobility of the esophagus. Understanding the basis for esophageal mobility may hold the key to developing methods to displace the structure during catheter ablation on the posterior left atrial wall and thus prevent esophageal injury.
